# Pulmonary Spindle Cell Carcinoma: A Rare Case Report

**DOI:** 10.7759/cureus.4737

**Published:** 2019-05-23

**Authors:** Salman Khan, Binita Dahal, Faraz Siddiqui, Kim J Norville, Apurwa Karki

**Affiliations:** 1 Internal Medicine, Guthrie Clinic/Robert Packer Hospital, Sayre, USA; 2 Internal Medicine - Critical Care, Guthrie Clinic/Robert Packer Hospital, Sayre, USA

**Keywords:** spindle cell, bronchoscopy

## Abstract

Spindle cell carcinoma is a rare pulmonary malignancy. The prognosis and treatments are unclear, with limited literature available. Here, we report a man who underwent bronchoscopy with an endobronchial biopsy after complaining of dyspnea on exertion. Computed tomography (CT) chest showed a 10 x 3 cm mass in the right upper lobe. Biopsy revealed spindle cell carcinoma, and he was referred to hematology for chemotherapy. He was lost to follow-up recently.

## Introduction

Sarcomatoid carcinoma of the lung is a rare, high-grade, poorly differentiated, non-small cell cancer. The incidence of sarcoma is 0.3% to 1.3% of all lung cancers [1-2]. According to the WHO classification, there are five histological variants of sarcomatoid carcinoma, among them is spindle cell carcinoma of the lung with rare histological subtypes [3]. A retrospective study of the 718 cases showed that spindle cell cancer accounted for 0.4% of all lung malignancy [4].

Spindle cell carcinoma consists of a pure population of spindle cells, which frequently involves the periphery of the lung [4]. Endobronchial spindle cell carcinoma is extremely rare and only a few cases of endobronchial leiomyosarcoma have been reported [5]. There have been limited cases of endobronchial spindle cell carcinomas. Here, we describe the case of an elderly male patient with incidental findings of endobronchial spindle cell carcinoma.

## Case presentation

An 85-year-old male presented to the emergency department of our hospital with progressive dyspnea for one week along with dry cough and progressive bilateral leg swelling. He was noted to be confused at the time of the presentation, unable to answer to place and time. The patient had a known history of ischemic cardiomyopathy, complete heart block with the placement of a permanent pacemaker, significant smoking history of 100 pack-years, and chronic obstructive pulmonary disease (COPD). On presentation, his vital signs revealed tachypnea with a respiratory rate of 40 a minute, along with diffuse bilateral crackles on lung auscultation and bilateral lower extremity edema.

**Figure 1:**
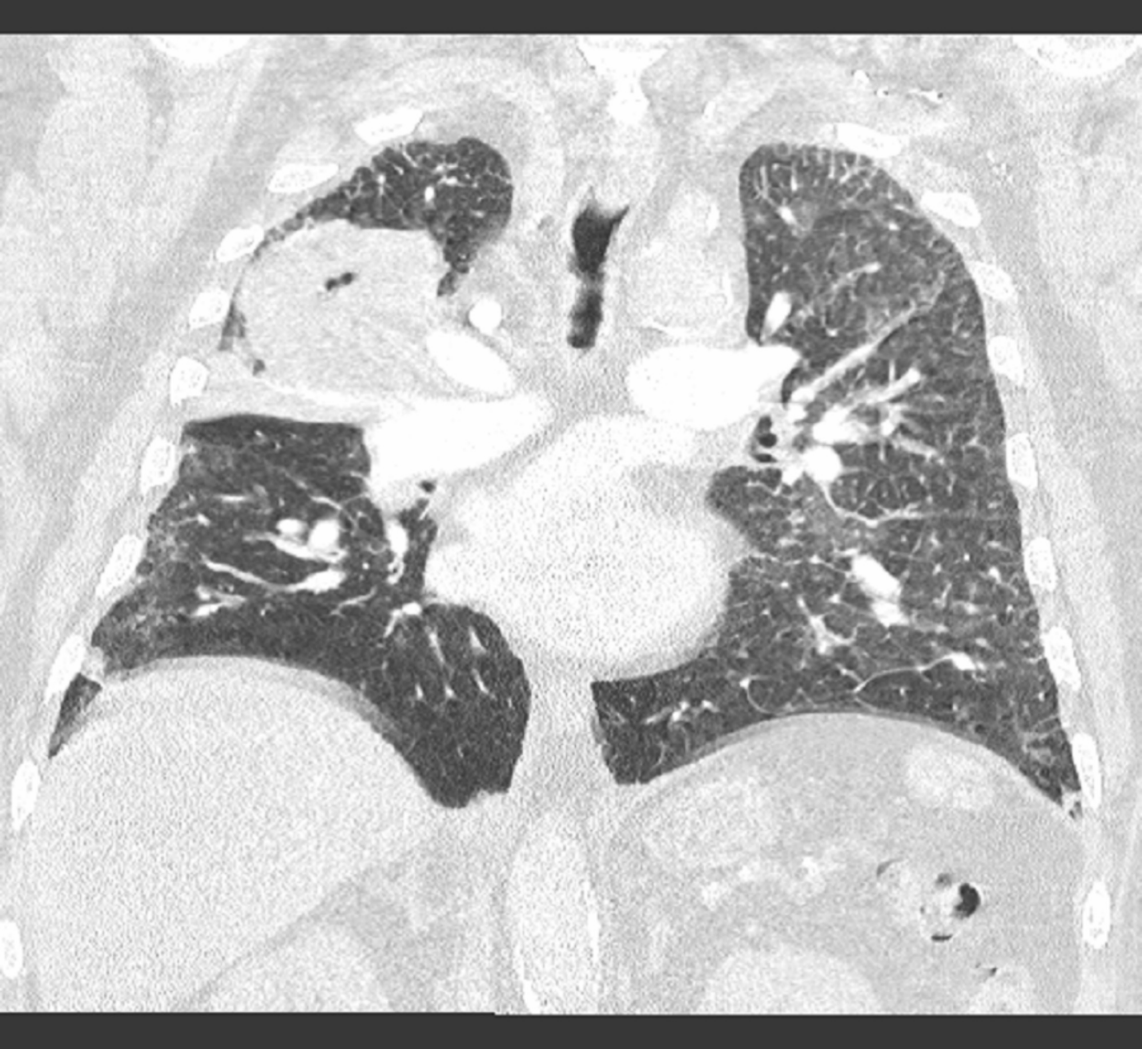
Coronal View of Computed Tomography Chest

**Figure 2:**
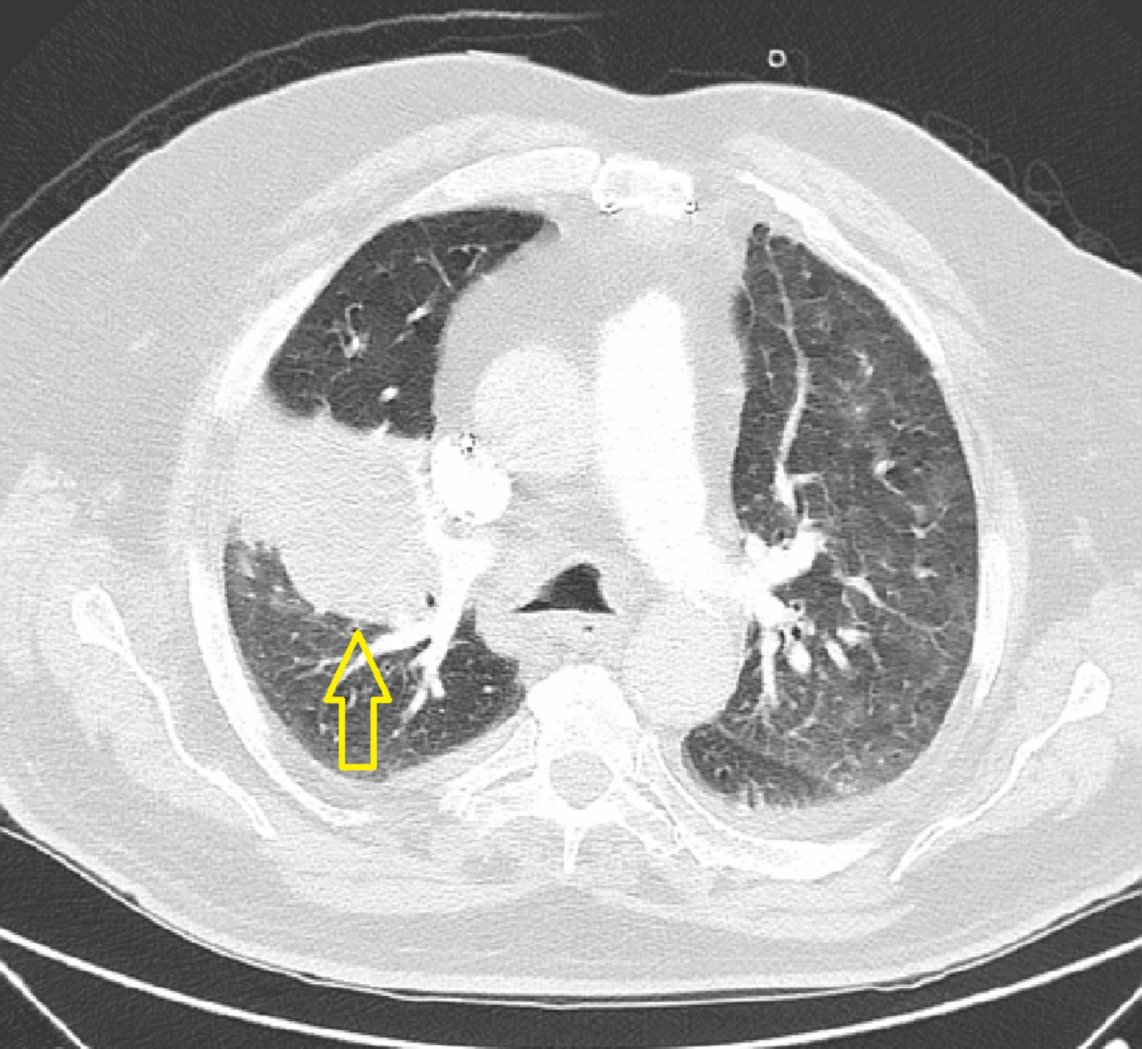
Transverse View of Computed Tomography Chest

His laboratory investigation was remarkable for a leukocyte count of 13.8 x 103/uL (normal = 4-11 x 103/uL), arterial blood gas showed acute hypoxia and hypercarbia with pH 7.29 (reference range: 7.35 - 7.45), PCO_2_ 66 mm of Hg (reference range: 35 - 40), and PO_2_ 75 mm of Hg (reference range: 80 - 100). Chest X-ray showed bilateral pleural effusions, increased interstitial markings, and a right upper lung zone opacity. Computed tomography (CT) of the chest was performed to characterize the nature of the opacity, which showed a 10 x 8 centimeter mass in the right upper lobe of the lung with no visible air bronchograms (Figure 1, Figure 2).

He was admitted to the medical intensive care unit (ICU) and managed with intravenous furosemide and non-invasive bilevel positive pressure ventilation, which resulted in a significant improvement in his respiratory symptoms.

**Figure 3:**
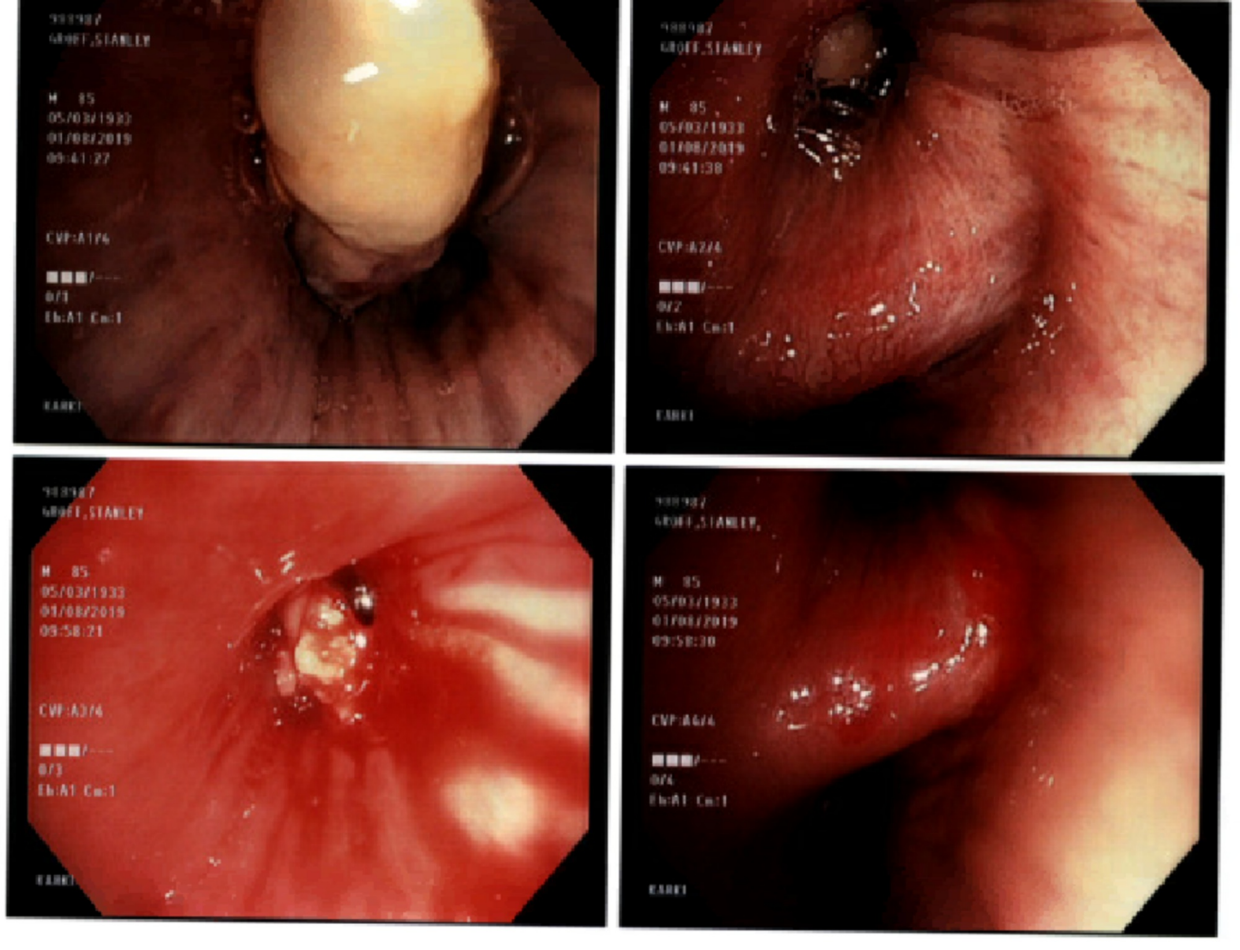
Bronchoscopic View of Tumor

**Figure 4:**
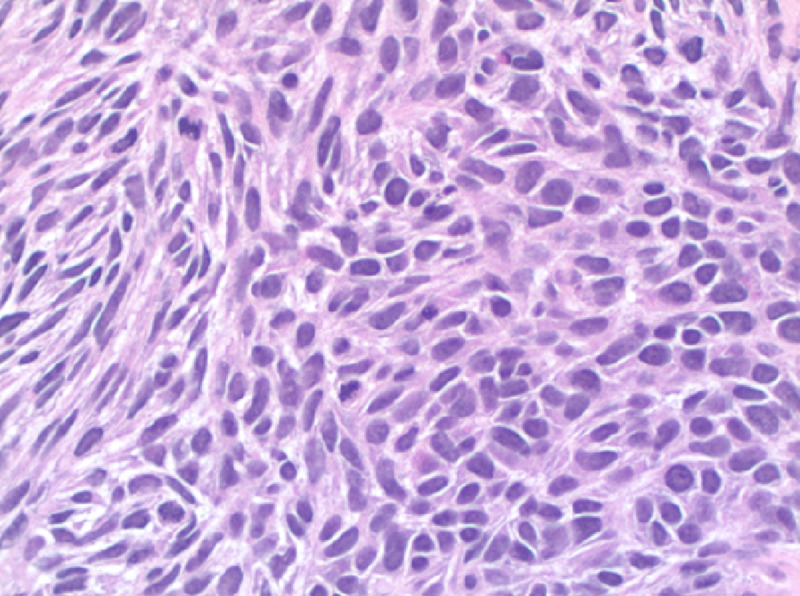
High-Grade Spindle Cell Sarcoma with Pleomorphic Spindle Cells and Increased Mitoses

The patient underwent fiberoptic flexible bronchoscopy to evaluate the mass. This revealed a near-complete occlusion of the right upper lobe bronchus with an exophytic fungating mass (Figure 3). Endobronchial biopsies were performed and suggestive that the patient had non-epithelial cancer originating from the endobronchial mucosa, with microscopic examination suggestive of spindle cell sarcoma (Figure 4).

Microscopic examination of the biopsied tissue showed extensive necrotic spindle cell sarcoma. Immunohistochemical stain for vimentin, CD99, and BCL-2 was positive, along with negative pan-keratin, CK56, p63, synaptophysin, chromogranin, and CD34. The patient was referred to hematology-oncology and radiation therapy. He completed 20 treatments of radiation therapy and was found to have an interval decrease in the size of the tumor on repeat imaging.

## Discussion

There have been no data available on the prognosis of endobronchial spindle cell cancer as such; however, as it is described under sarcomatoid cancer, its prognosis is poor. Overall survival in a patient with spindle cell cancer depends on the age of the patient, the location of cancer, and staging at the time of diagnosis of cancer [1]. Spindle cell carcinoma is more common in men than in women, with an approximate ratio of 2.7:1 and a median age at diagnosis of 62 years [1]. Different hypotheses have been postulated by different authors in regards to the origin of spindle cell cancer. The accepted hypothesis is that spindle cell cancer results from the metaplasia of neoplastic cells [2].

Sarcomatoid carcinomas are of the following histological sub-types: pleomorphic, spindle cell carcinoma, giant cell carcinoma, carcinosarcoma, and pulmonary blastoma [3]. These carcinomas are sometimes described as pulmonary pleomorphic cell carcinoma [6]. Our case is rare in that it is of non-epithelial origin and, hence, not a carcinoma according to the histological examination.

Initial presentation may be chest pain, cough, shortness of breath, or hemoptysis. Computed tomography demonstrates that a peripheral cavitary lesion is common, however, there may be a collapse of certain lung segments if there is the presence of an endobronchial lesion [6]. Spindle cell cancer is strongly associated with cigarette smoking, as our patient had a significant smoking history as well [7]. In a clinicopathologic review of 78 cases of pleomorphic carcinoma of the lung, 18% of patients had incidental findings on chest X-ray and spindle cell carcinoma was most frequently associated with giant cell carcinoma [1]. Most of the spindle cell cancers arise in the upper lobe of the peripheral lung [8].

Immunohistochemical markers are highly positive for pan-cytokeratin, vimentin, Ki67, and negative for other markers like CK5/6, p63 epithelial markers, synaptophysin, and chromogranin [4]. Other endobronchial lesions, such as leiomyomas, showed more consistent staining, with smooth muscle markers such as smooth muscle actin, desmin, and smooth muscle myosin heavy chain [5]. Treatment depends on the staging of cancer and may include surgical resection, chemotherapy, and radiation therapy. Among those patients with surgery, the recurrence rate was 30% [8].

## Conclusions

Pulmonary spinal cell carcinoma is a rare and aggressive malignancy. Our patient was an active smoke who never received a low-dose lung CT for cancer screening. He was admitted to the ICU and was found to have a right upper lobe mass. The biopsy confirmed the diagnosis, and he was referred to radiation therapy. Lung cancer screening should be reinforced in clinical practice and may have, perhaps, detected his mass at an earlier stage as compared to his late-stage presentation. Emphasis should be placed on smoking cessation and lung cancer screenings.
